# Adipocyte-derived exosomes promote lung cancer metastasis by increasing MMP9 activity via transferring MMP3 to lung cancer cells

**DOI:** 10.18632/oncotarget.18737

**Published:** 2017-06-27

**Authors:** Jiaoli Wang, Yilei Wu, Jufeng Guo, Xuefeng Fei, Lei Yu, Shenglin Ma

**Affiliations:** ^1^ Department of Respiratory Medicine, Nanjing Medical University, Affiliated Hangzhou Hospital (Hangzhou First People’s Hospital), Hangzhou, China; ^2^ Department of General Surgery, Ruian People’s Hospital, Wenzhou, China; ^3^ Department of Breast Surgery, Nanjing Medical University, Affiliated Hangzhou Hospital (Hangzhou First People’s Hospital), Hangzhou, China; ^4^ Institute of Immunology, Zhejiang University School of Medicine, Hangzhou, China; ^5^ Laboratory of Cancer Epigenetics, Department of Medical Oncology, Biomedical Research Center, Sir Run Run Shaw Hospital, School of Medicine, Zhejiang University, Hangzhou, China; ^6^ Department of Oncology, Nanjing Medical University, Affiliated Hangzhou Hospital (Hangzhou First People’s Hospital), Hangzhou, China

**Keywords:** exosomes, adipocytes, lung cancer, MMP3, MMP9

## Abstract

Obesity is involved in tumor progression. However, the corresponding mechanisms remain largely unknown. Here, we report that adipocytes increase the invasive ability of tumor cells by producing exosomes with a high level of MMP3. Compared with 3T3-L1 cells, 3T3-L1 adipocytes are enriched in MMP3 protein and can transfer MMP3 to 3LL lung cancer cells. Then, MMP3 activates MMP9 activity in 3LL cells and promotes invasion *in vitro* and *in vivo* via MMP9. Furthermore, MMP3 protein levels in lung tumor tissues from obese patients are increased compared with those of non-obese patients. In addition, MMP3 protein levels are positively correlated with MMP9 activity in tumor tissues. Therefore, our results reveal a novel mechanism in the adipocyte-derived exosome-mediated promotion of lung tumor metastasis, which extends our knowledge regarding obesity and tumor progression.

## INTRODUCTION

Lung cancer is the leading cause of cancer death among males in worldwide and has surpassed breast cancer as the leading cause of cancer death among females in more developed countries [1]. Despite advances in diagnosis, staging, surgical techniques, and neoadjuvant chemoradiotherapy over the last decade, the mortality rate of lung cancer remains high, and the 5-year survival rate is only 15% [2, 3]. Metastasis is the main cause of lung cancer therapy failure. Therefore, a further understanding of the mechanisms of lung cancer metastasis is urgently needed.

Obesity has been demonstrated to be positively associated with tumor metastasis [4–6]. Obesity contributes to ovarian cancer metastatic success by increasing lipogenesis, enhancing vascularity, and decreasing the infiltration of M1 macrophages [7]. The mature adipocyte-stimulated expression of CCL19 and CCL21 in lymphatic endothelial cell, and expression of their receptor CCR7 in melanoma cells, which contributes to increased lymph node metastasis of melanoma in high-fat diet-fed mice [8]. Human adipose tissue-derived stem cells promote breast cancer cell invasion in a CCL5-depedent fashion [9]. Obesity is also implicated in the increased metastasis of lung cancers. Mice fed a high-fat diet exhibit a significantly increased number and size of lung metastases compared with those fed a control diet [10]. Leptin, an adipocyte-derived cytokine associated with obesity, effectively enhances metastasis of the human lung cancer A549 cell line [11]. However, the mechanism by which obesity promotes lung cancer cell metastasis has yet to be explored.

Exosomes, which are 50 to 150 nm in diameter, are vesicles with a lipid bi-layer membrane structure that are released by a variety of live cells. Exosomes are released into the extracellular milieu when multi-vesicular bodies fuse with the cell membrane [12, 13]. When shuttled from a donor cell, an exosome can transfer a broad array of biological contents, including functional mRNAs, miRNAs, DNA fragments, lipids and proteins, to recipient cells [14]. Exosomes are a mediator of tumor metastasis. Exosomes from activated T cells promote tumor metastasis through upregulation of MMP9 protein levels in tumor cells via Fas signaling [15]. Exosomes release miR-126a from MDSC in response to doxorubicin treatment to promote lung metastasis [16]. Whether exosomes from adipocytes are involved in tumor metastasis remains unknown.

In this study, we evaluated the effect of 3T3-L1 adipocyte-derived exosomes (3T3-A-EXO) on murine 3LL Lewis lung cancer cells. We found that 3T3-A-EXO promotes 3LL tumor cell invasion *in vitro* through increasing MMP9 activity but not protein levels. In addition, 3T3-A-EXO is enriched in MMP3 mRNA and protein, which could be transferred into 3LL tumor cells. After transfer, MMP3 exhibited a robust ability to activate MMP9 and promote tumor metastasis *in vivo*.

## RESULTS

### Characterization of 3T3-A-EXO

**Figure 1:**
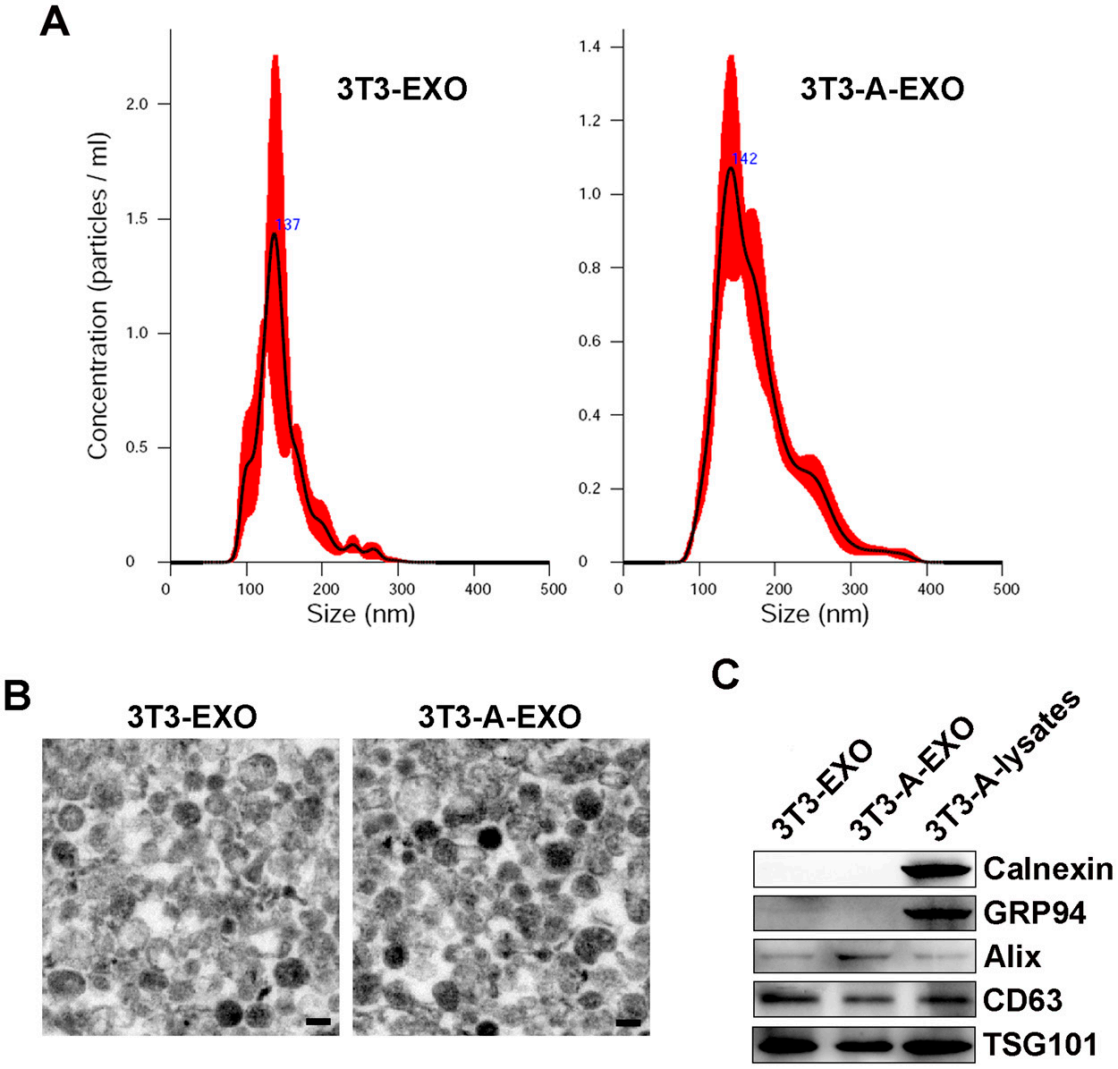
Characterization of 3T3-EXO and 3T3-A-EXO **(A)** Assessment of size distribution of 3T3-EXO and 3T3-A-EXO by nanoparticle tracking analysis technology. **(B)** Morphology of 3T3-EXO and 3T3-A-EXO under electron microscopy. Scale bar = 100 nm. **(C)** Western blot detection of Calnexin, GRP94, Alix, CD63 and TSG101 in 3T3-EXO and 3T3-A-EXO. The data are representative of three independent experiments.

Exosomes from 3T3-L1 cells (3T3-EXO) or 3T3-A-EXO isolated by sequential ultracentrifugation were first evaluated by nanoparticle tracking analysis technology, showing a vesicle population with a peak at 137 or 142 nm for 3T3-EXO or 3T3-A-EXO, respectively (Figure 1A). Electron analysis revealed a population of vesicles with a bi-layer membrane structure ranging from 50 to 150 nm in diameter (Figure 1B). The presence of several exosomal markers, such as CD63, TSG101 and Alix, in both exosomes were confirmed by Western blot analysis (Figure 1C). The absence of endoplasmic reticulum-residing protein GRP94 and Calnexin indicated the purity of exosomes (Figure 1C).

### 3T3-A-EXO promoted 3LL tumor cell invasion *in vitro*

**Figure 2:**
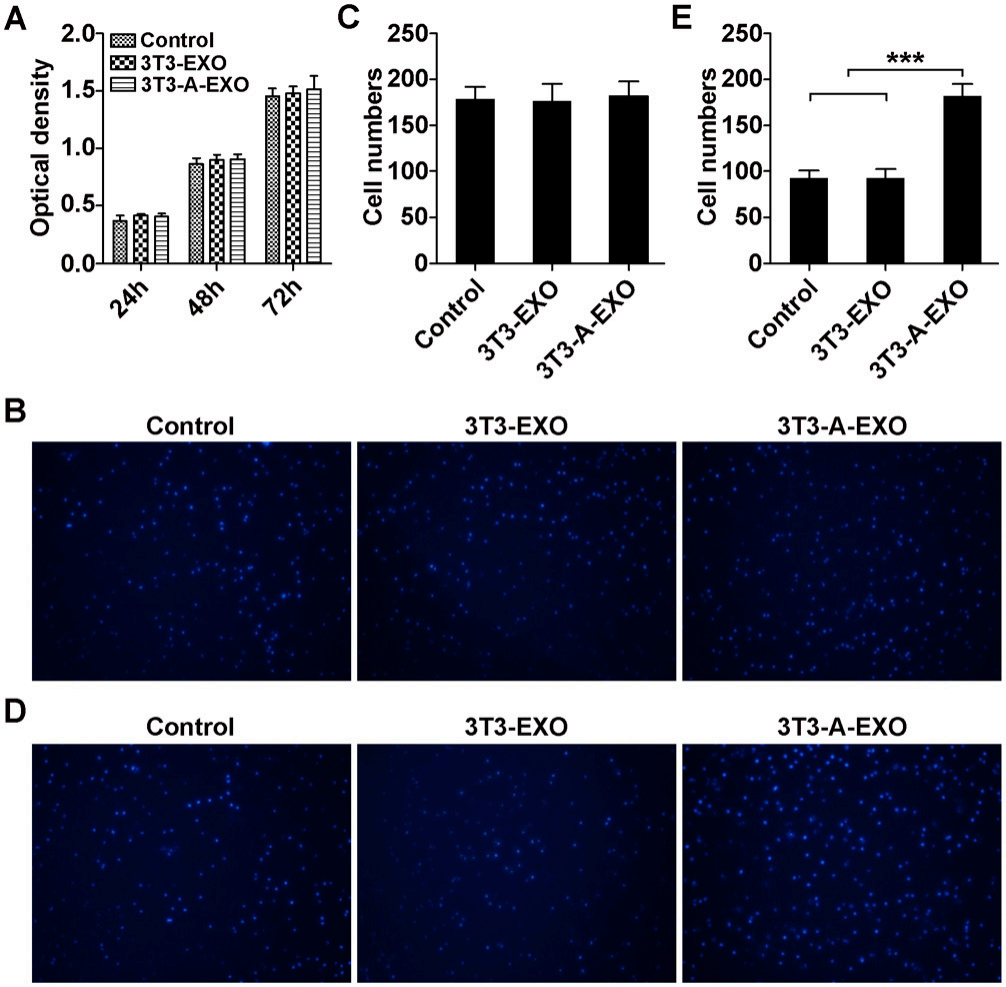
3T3-A-EXO promoted 3LL tumor cell invasion *in vitro* **(A)** 3LL cells were pretreated with 30 μg/ml 3T3-EXO or 3T3-A-EXO for 4 h. Then, 3LL cell growth was detected at 24, 48 and 72 h by CCK-8 assay (n = 3). **(B)** 3LL cells were treated with 30 μg/ml 3T3-EXO or 3T3-A-EXO for 4 h. Then, the cells were plated in the top chamber of a Transwell plate. Twenty-four hours later, the number of cells on the bottom of the Transwell filter was imaged and quantified. **(C)** Statistical analysis of result B (n = 5). **(D)** 3LL cells were treated with 30 μg/ml 3T3-EXO or 3T3-A-EXO for 4 h. Then, the cells were plated in the top chamber precoated with 50 μl of Matrigel. Forty-eight hours later, the number of cells on the bottom of the Transwell filter was imaged and quantified. **(E)** Statistical analysis of result **(D)** (n = 5). **(A, C, E)** Results are presented as the mean ± SEM of three independent experiments. **(B, D)** One representative image out of five is presented. *P*-values were generated by one-way ANOVA followed by Tukey-Kramer multiple comparisons test; ***p< 0.001. Control indicates 3LL cells treated with PBS.

First, we investigated whether adipocyte-derived exosomes affect the proliferation of 3LL tumor cells *in vitro*. Briefly, 3LL cells were pretreated with 3T3-EXO or 3T3-A-EXO for 4 h, and 3LL cell growth was detected. As shown in Figure 2A, both exosomes could not affect the growth of 3LL cells. In addition, 3T3-EXO or 3T3-A-EXO showed no effect on 3LL cell apoptosis (Supplementary Figure 1). Therefore, adipocyte-derived exosomes can not affect the proliferation of 3LL tumor cells *in vitro*. To determine whether adipocyte-derived exosomes played a role in 3LL cell migration or invasion, 3T3-EXO- or 3T3-A-EXO-pretreated 3LL cells were placed in the top chamber in serum-free media, whereas the bottom chamber contained media with 20% FCS. Pretreatment of both exosomes did not cause more cells migrate to the bottom chamber in the migration assay (Figure 2B, 2C). However, in the invasion assay, more 3T3-A-EXO- but not 3T3-EXO-pretreated 3LL cells invaded into the bottom chamber (Figure 2D, 2E). These results indicate that adipocyte-derived exosomes play a role in enhancing the invasive ability of 3LL cells *in vitro*.

### 3T3-A-EXO promoted 3LL tumor cell invasion through MMP3-mediated increases in MMP9 activation

**Figure 3:**
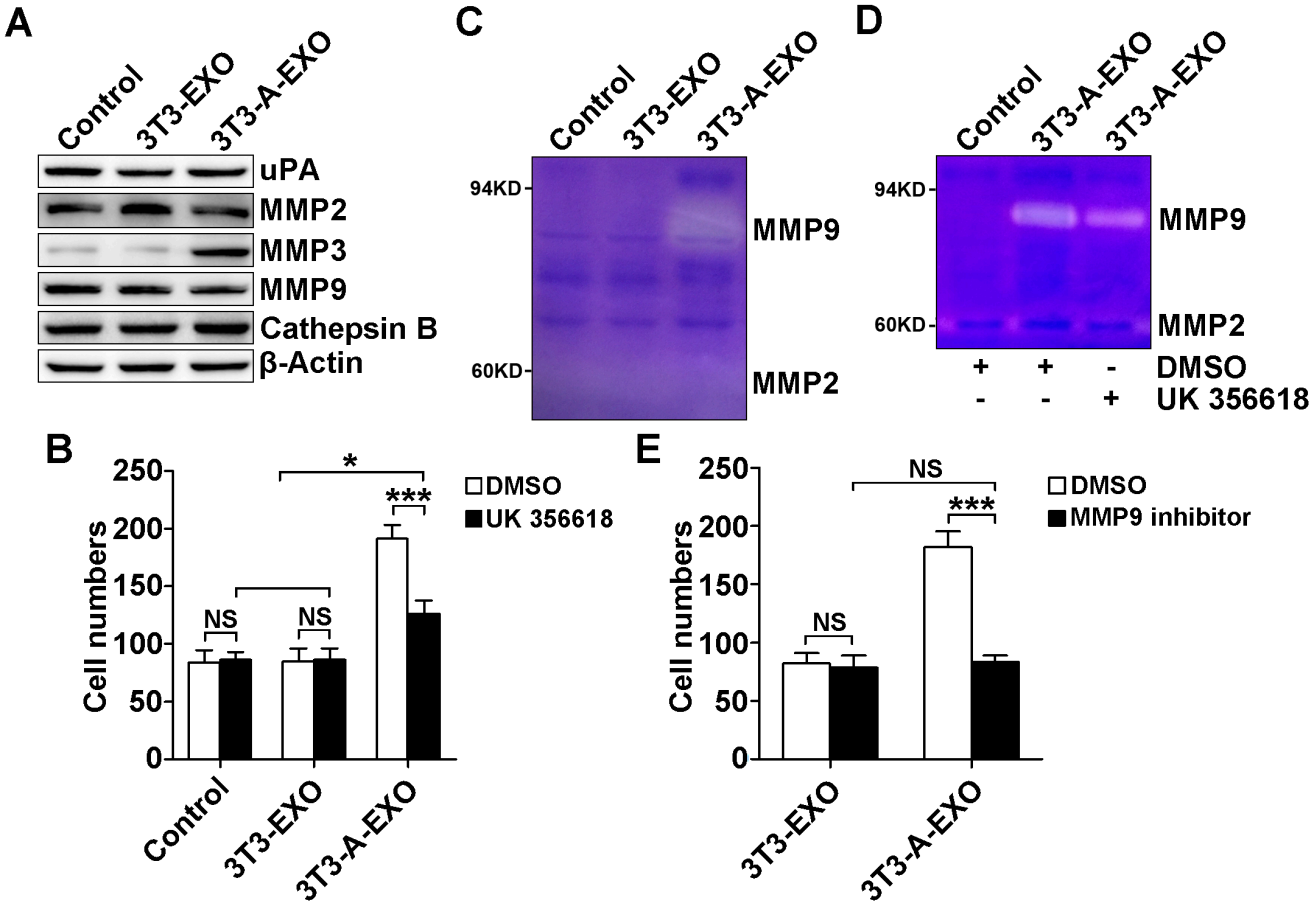
3T3-A-EXO promoted 3LL tumor cell invasion through MMP3-mediated increase in MMP9 activation **(A)** 3LL cells were treated with 30 μg/ml 3T3-EXO or 3T3-A-EXO for 4 h, and then the protein levels of uPA, MMP2, MMP3, MMP9 and cathepsin B were detected by Western blot. **(B)** After the treatment with 30 μg/ml 3T3-EXO or 3T3-A-EXO for 4 h, 3LL cells were collected and pre-treated with 20 nM UK 356618 for 2 h. Then, the invasive ability of these cells was measured by an *in vitro* invasive assay (n = 5). **(C)** 3LL cells were treated with 30 μg/ml 3T3-EXO or 3T3-A-EXO for 4 h. Then, cells were collected and cultured in serum-free RPMI 1640 media for an additional 24 h. MMP2 and MMP9 activity in the supernatants was detected by gelatin zymography. **(D)** 3LL cells were treated with 30 μg/ml 3T3-A-EXO for 4 h, and then the cells were collected and cultured in serum-free RPMI 1640 media in the presence of 10 nM UK 356618 for an additional 24 h. MMP2 and MMP9 activity in the supernatants was detected by gelatin zymography. E, 3LL cells were treated with 30 μg/ml 3T3-EXO or 3T3-A-EXO with or without 10 nM MMP9 inhibitor for 4 h, and then the invasive ability of these cells was measured using an *in vitro* invasive assay (n = 5). **(A, C, D)** One representative of three independent experiments is presented. **(B, E)** Results are presented as the mean ± SEM of three independent experiments. **(B, D, E)** DMSO is solvent control. *P*-values were generated by Student’s *t*-test or one-way ANOVA followed by Tukey-Kramer multiple comparisons test; *p< 0.05; ***p< 0.001; NS, not significant. Control indicates 3LL cells treated with PBS.

MMP2, MMP3, MMP9, uPA and Cathepsin B play critical roles in tumor invasion and metastasis [17–20]. We examined uPA, MMP2, MMP3, MMP9 and Cathepsin B protein levels in 3LL cells treated with 3T3-EXO or 3T3-A-EXO by Western blot. Only MMP3 protein levels were increased in 3LL cells treated with 3T3-A-EXO (Figure 3A). To elucidate whether increased MMP3 was involved in the enhanced invasive ability of 3LL cells, exosome-treated 3LL cells were pretreated with the MMP3-specific inhibitor UK 356618, and then the invasive ability of 3LL cells was detected. After the treatment with the MMP3 inhibitor, the invasive ability of 3T3-A-EXO-treated 3LL cells was significantly inhibited, but that of 3T3-A-EXO-treated 3LL cells remained stronger than 3T3-EXO- or PBS-treated 3LL cells (Figure 3B). Given that MMP3 effectively activates MMP9 [21], we interrogated whether MMP3 increased the invasive ability of 3T3-A-EXO-treated 3LL cells through inducing MMP9 activation. As expected, increased MMP9 but not MMP2 activity was detected in 3T3-A-EXO- but not 3T3-EXO- or PBS-treated 3LL cells when examined by gelatin zymography (Figure 3C). Consistent with the invasive ability, MMP3 inhibitor treatment markedly reduced but did not completely abolish 3T3-A-EXO-induced MMP9 activity in 3LL cells (Figure 3D). To elucidate whether MMP3 increased the invasive ability of 3T3-A-EXO-treated 3LL cells through inducing MMP9 activation, exosome-treated 3LL cells were pretreated with the MMP9-specific inhibitor, and then the invasive ability of 3LL cells were determined *in vitro*. No difference in the invasive ability of 3T3-EXO- and 3T3-A-EXO-treated 3LL cells was noted after MMP9 inhibition (Figure 3E). Altogether, these results suggest that increased MMP3 in 3T3-A-EXO-treated 3LL cells enhances 3LL cell invasive ability through inducing MMP9 activation.

### 3T3-A-EXO transferred MMP3 to 3LL tumor cells

**Figure 4:**
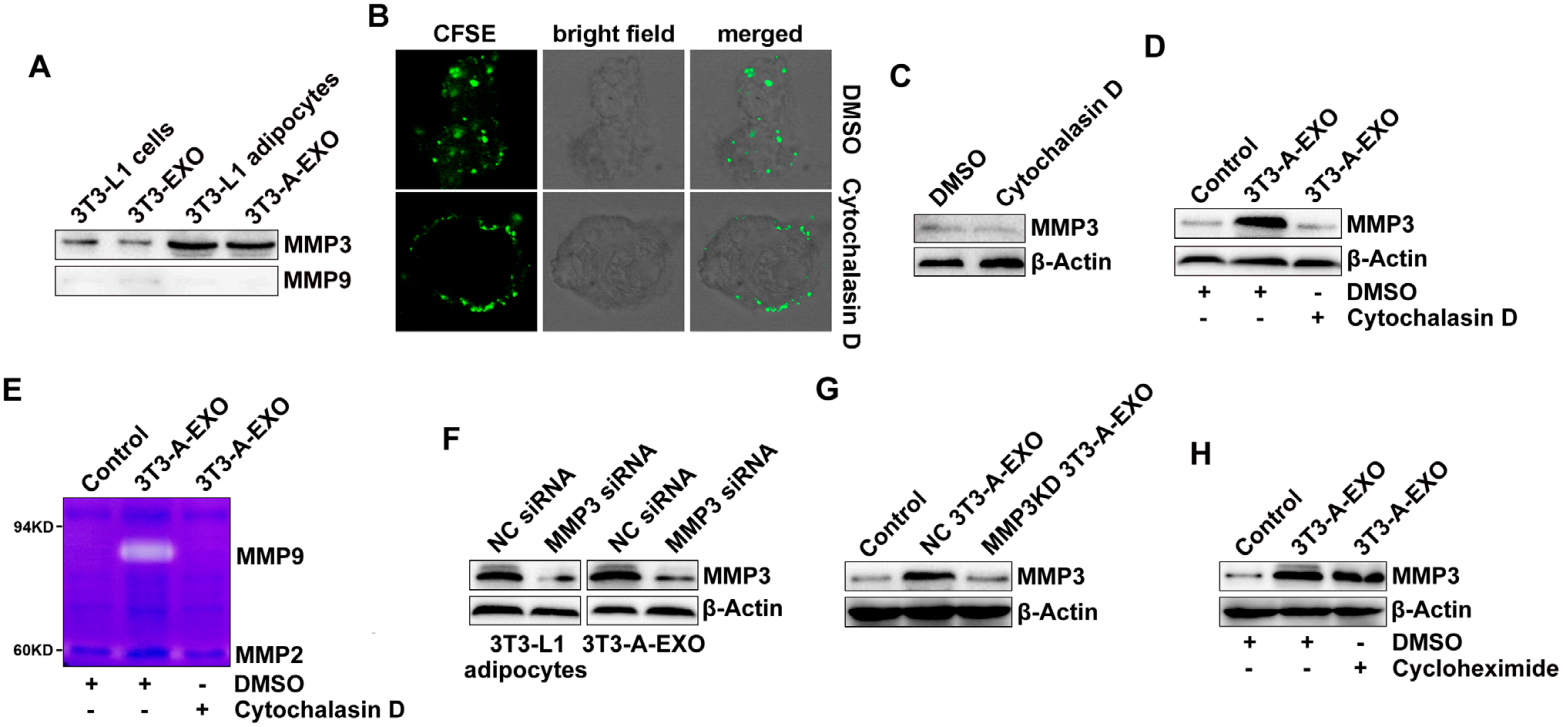
3T3-A-EXO transferred MMP3 to 3LL tumor cells **(A)** MMP3 and MMP9 protein levels in 20 μg 3T3-EXO, 3T3-A-EXO, 3T3-L1 or 3T3-L1 lysates were detected by Western blot. **(B)** 3LL cells were pre-treated with 10 μg/ml cytochalasin D for 30 min and then co-cultured with CFSE-labeled 3T3-A-EXO for 4 h. The uptake of 3T3-A-EXO by 3LL cells was detected by confocal microscopy. **(C, D)** After the pre-treatment with 10 μg/ml cytochalasin D for 30 min, 3LL cells were collected and re-cultured in fresh RPMI 1640 media containing 10% FBS **(C)** or treated with 30 μg/ml 3T3-A-EXO **(D)** for 4 h. MMP3 protein levels in these cells were detected by Western blot. **(E)** After the pre-treatment with 10 μg/ml cytochalasin D for 30 min, 3LL cells were treated with 30 μg/ml 3T3-A-EXO for 4 h, and then the cells were collected and cultured in serum-free RPMI 1640 media for an additional 24 h. The MMP2 and MMP9 activity in the supernatants was detected by gelatin zymography. **(F)** 3T3-L1 adipocytes were transfected with MMP3 siRNA or negative control (NC) siRNA for 24 h, and then MMP3 protein levels in the cells or 3T3-A-EXO were detected by Western blot. **(G)** 3LL cells were treated with 30 μg/ml NC 3T3-A-EXO or MMP3KD 3T3-A-EXO for 4 h, and then MMP3 protein levels were detected by Western blot. **(H)** 3LL cells were treated with 30 μg/ml 3T3-A-EXO in the presence of 10 μg/ml cycloheximide for 4 h, and then MMP3 protein levels were detected by Western blot. The data are representative of three independent experiments. Control indicates 3LL cells treated with PBS. **(C-D, E, H)** DMSO is solvent control.

Next, we wanted to determine how 3T3-A-EXO increased MMP3 protein levels in 3LL cells. When detected by Western blot, we found that MMP3 but not MMP9 protein was enriched in 3T3-L1 adipocytes and 3T3-A-EXO (Figure 4A). In addition, MMP3 and MMP9 proteins were minimally detected in 3T3-L1 cells and 3T3-EXO (Figure 4A). Microvesicles from A549 tumor cells contained a high level of EGFR and could transfer EGFR to endothelial cells [22]. Therefore, we hypothesized that 3T3-A-EXO increased MMP3 protein levels in 3LL cells through transferring MMP3 to 3LL cells. We inhibited exosome uptake by 3LL cells by pre-treating 3LL cells with cytochalasin D and confirmed the inhibitory effect (Figure 4B). Furthermore, treatment of 3LL cells with cytochalasin D caused no change in MMP3 protein levels (Figure 4C). After inhibition of exosome uptake by cytochalasin D, 3T3-A-EXO did not further increased MMP3 protein levels in 3LL cells (Figure 4D). In addition, increased MMP9 activity could also not be detected (Figure 4E). To further confirm 3T3-A-EXO transfer of MMP3 to 3LL cells, we knocked down MMP3 in 3T3-L1 adipocytes by siRNA and confirmed the knocked down effect of MMP3 in both 3T3-L1 adipocytes and 3T3-A-EXO (Figure 4F). Exosomes from negative control (NC)-siRNA transfected 3T3-L1 adipocytes (NC 3T3-A-EXO) obviously increased MMP3 levels in 3LL cells, whereas exosomes from MMP3-siRNA transfected 3T3-L1 adipocytes (MMP3KD 3T3-A-EXO) did not increase these levels (Figure 4G). To exclude the possibility that 3T3-A-EXO increased MMP3 in 3LL cells by inducing *de novo* MMP3 production, 3LL cells were treated with 3T3-A-EXO in the presence of cycloheximide. Cycloheximide showed no effect on the 3T3-A-EXO-mediated MMP3 increase in 3LL cells (Figure 4H). Furthermore, we did not detect the increase of *MMP3* mRNA expression in 3T3-A-EXO-treated 3LL cells (Supplementary Figure 2). Together, these results demonstrate that 3T3-A-EXO increased MMP3 protein levels in 3LL cells through a protein-transfer mechanism.

### 3T3-A-EXO promotes 3LL tumor cell metastasis *in vivo* via the MMP3/MMP9 axis

**Figure 5:**
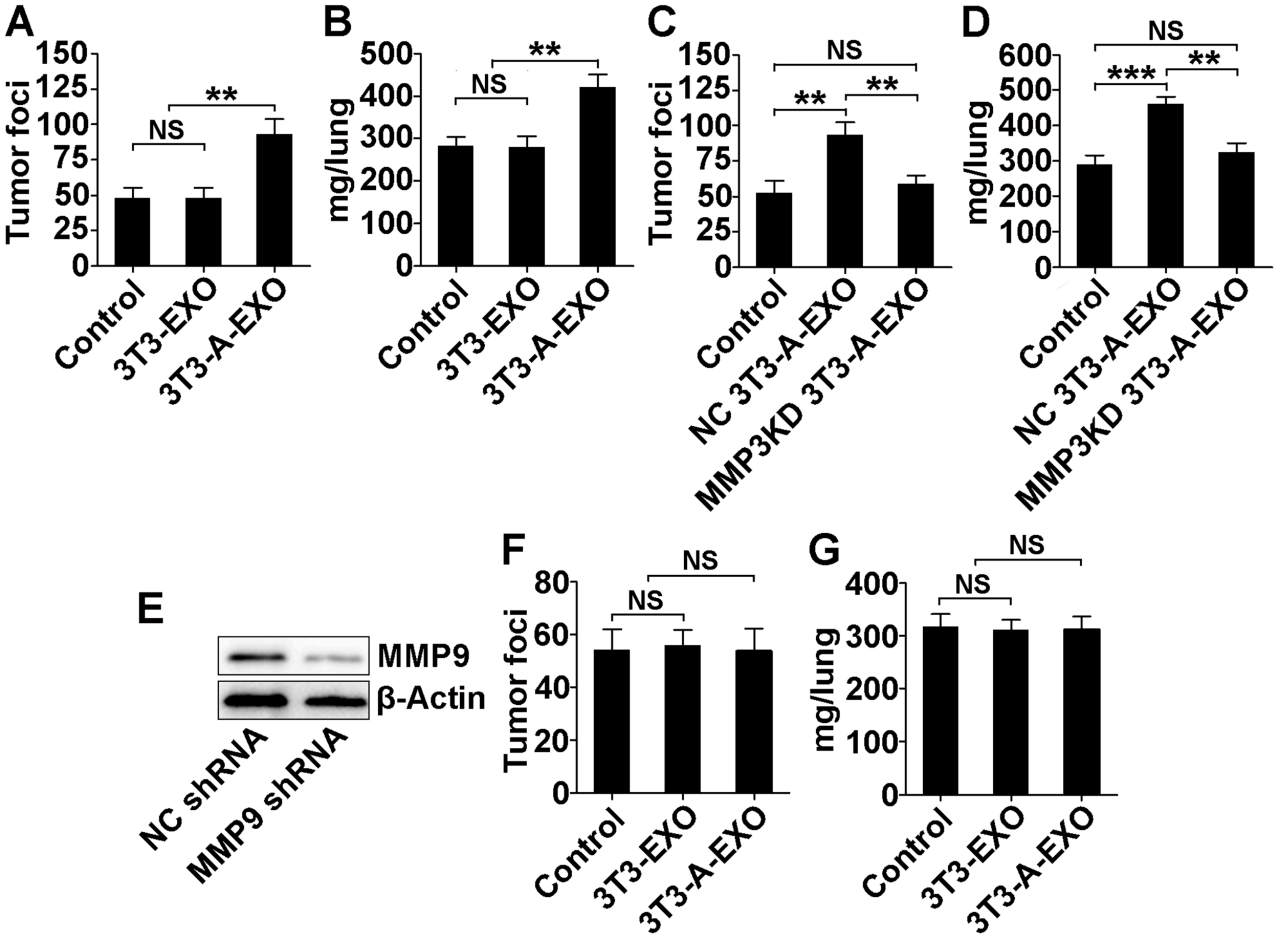
3T3-A-EXO promotes 3LL tumor cell metastasis *in vivo* via MMP3/MMP9 axis **(A, B)** 3LL cells were treated with 30 μg/ml 3T3-EXO or 3T3-A-EXO for 4 h. Then, 1×10^6^ tumor cells were intravenously injected into mice, and these mice were euthanized 15 days later. Lung tumor foci were statistically analyzed (n = 6) **(A)**. The weights of lungs were statistically analyzed (n = 6) **(B)**. **(C, D)** 3LL cells were treated with 30 μg/ml NC 3T3-A-EXO or MMP3KD 3T3-A-EXO for 4 h. Then, 1×10^6^ tumor cells were intravenously injected into mice, and these mice were euthanized 15 days later. Lung tumor foci were statistically analyzed (n = 6) **(C)**. The weights of lungs were statistically analyzed (n = 6) **(D)**. **(E)** MMP9 protein levels in 3LL cells stably transfected with NC or MMP9 shRNA were detected by Western blot. **(F, G)** 3LL cells stably transfected with MMP9 shRNA were treated with 30 μg/ml 3T3-EXO or 3T3-A-EXO for 4 h. Then, 1×10^6^ tumor cells were intravenously injected into mice, and these mice were euthanized 15 days later. Lung tumor foci were statistically analyzed (n = 6) **(F)**. The weights of lungs were statistically analyzed (n = 6) **(G)**. **(A-D, F, G)** The results are shown as the mean ± SEM of three independent experiments. **(E)** One representative of three independent experiments is shown. *P*-values were generated by one-way ANOVA, followed by Tukey-Kramer multiple comparisons test; **p< 0.01; ***p< 0.001; NS, not significant. Control indicates 3LL cells treated with PBS in **(A-D)** and 3LL cells with MMP9 KD treated with PBS in **(F, G)**, respectively.

To gain further insight into the effect of adipocyte-derived exosomes on lung cancer metastasis, we intravenously injected exosome-treated 3LL cells into mice and then detected tumor lung metastasis. Compared with lungs from mice that received 3T3-EXO- or PBS-treated 3LL cell transfer, lungs from mice that received 3T3-A-EXO-treated 3LL cell transfer exhibited significantly increased tumor foci (Figure 5A). We also weighed the lungs of each group. Lungs from mice subject to 3T3-A-EXO-treated 3LL cell transfer weighed more, indicating the most severe tumor burden (Figure 5B). We also used human lung adenocarcinoma A549 cells in the *in vivo* metastasis model and found the similar results with 3LL cells (Supplementary Figure 3A, 3B). Both tumor foci in the lungs and weights of the lungs from mice that received MMP3KD 3T3-A-EXO-treated 3LL cell transfer were reduced compared with mice that received NC 3T3-A-EXO-treated 3LL cell transfer (Figure 5C, 5D), suggesting that MMP3 promoted 3T3-A-EXO-mediated 3LL tumor cell metastasis *in vivo*. To determine whether MMP9 was also involved in this process, we established a 3LL cell line with stable MMP9 knockdown. Transfection with a plasmid containing MMP9 shRNA markedly inhibited MMP9 protein levels in 3LL cells (Figure 5E). After MMP9 knockdown, lungs from mice that received 3T3-EXO- or 3T3-A-EXO-treated 3LL cell transfer exhibited similar tumor foci and weights (Figure 5F, 5G). Together, these data indicate both MMP3 and MMP9 are involved in the 3T3-A-EXO-mediated increase in 3LL tumor cell metastasis *in vivo*.

### Lung tumor tissues from obese tumor patients exhibited increased MMP3 protein levels and MMP9 activity

**Figure 6:**
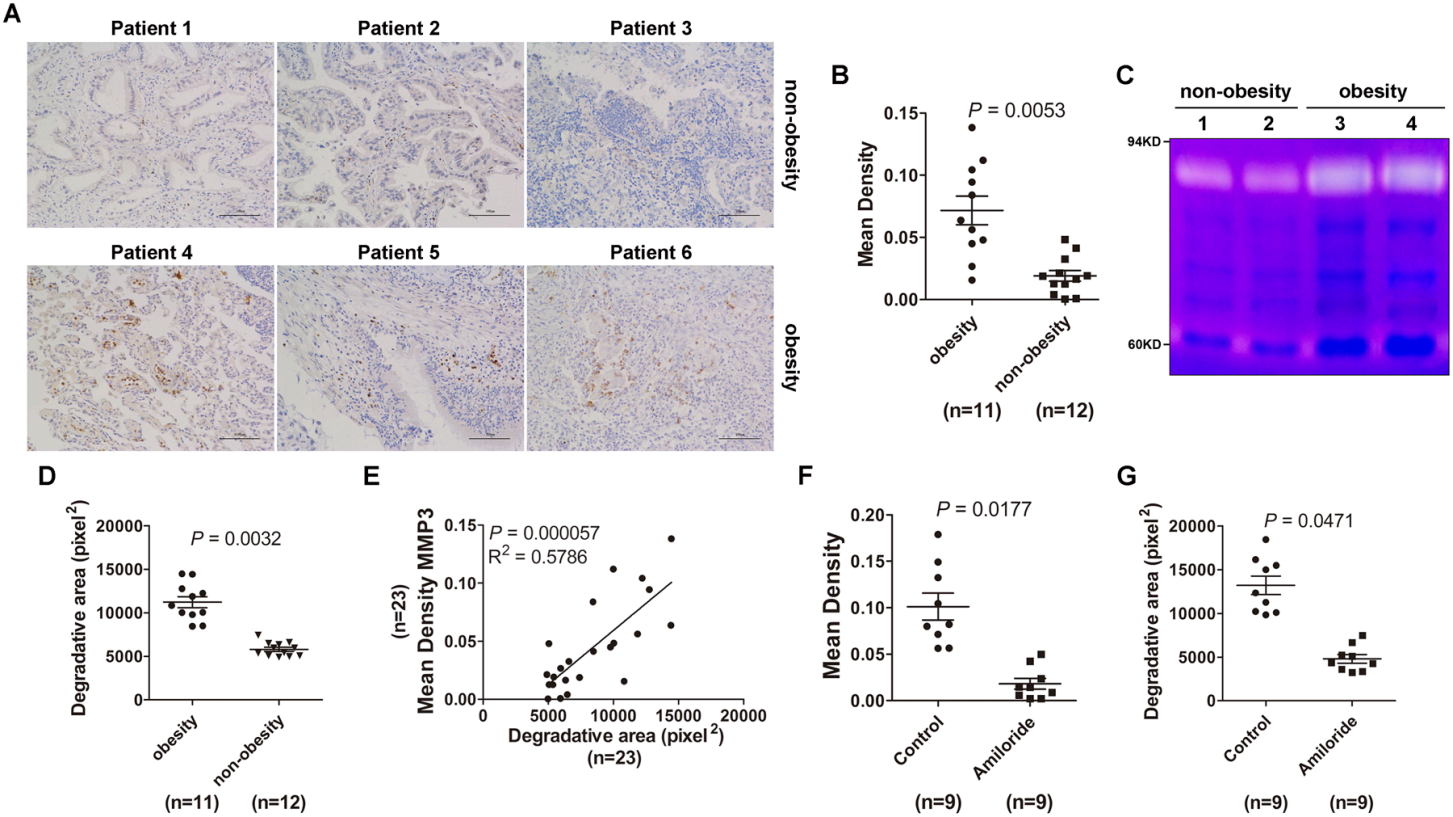
Lung tumor tissues from obese tumor patients exhibited increased MMP3 protein levels and MMP9 activity **(A)** MMP3 protein levels in tumor tissues from lung cancer patients were detected by IHC. The data are three representative from the results of obese or non-obese lung cancer patients. Scale bar = 100 μm. **(B)** Five images were randomly captured from each IHC section. Images were analyzed by ImageJ 1.8.0, and the integrated optical density of MMP3 staining was statistically analyzed. **(C)** MMP2 and MMP9 activity in tumor tissues from lung cancer patients was detected by gelatin zymography. The data are three representative from the results of obesity or non-obesity lung cancer patients. **(D)** The area degraded by MMP9 was quantified using ImageJ 1.8.0 and statistically analyzed. **(E)** The correlation between MMP3 protein level and MMP9 activity in tumor tissues from lung cancer patients was analyzed by Spearman correlation analysis using GraphPad Prism 5. **(F)** MMP3 protein levels in tumor tissues from lung cancer patients with or without amiloride treatment were detected by IHC. Five images were randomly captured from each IHC section. Images were analyzed by ImageJ 1.8.0, and the integrated optical density of MMP3 staining was statistically analyzed. **(G)** MMP2 and MMP9 activity in tumor tissues from lung cancer patients with or without amiloride treatment was detected by gelatin zymography. The area degraded by MMP9 was quantified using the ImageJ 1.8.0 and statistically analyzed. **(B, D, F, G)** Results are presented as the mean ± SEM. *P*-values were generated by Student’s *t*-test. Control indicates patients without amiloride treatment.

Finally, we want to know whether obesity could affect MMP3 protein levels and MMP9 activity in lung tumor tissues. Immunohistochemistry results revealed that MMP3 protein levels in lung tumor tissues from obese tumor patients were increased compared with non-obese tumor patients (Figure 6A, 6B). When equal amounts of lung tumor tissue lysates were loaded for the gelatin zymography test, we detected increased MMP9 activities in lung tumor tissues from obese tumor patients compared with non-obese tumor patients (Figure 6C, 6D). In addition, MMP3 protein levels were positively correlated with MMP9 activity (Figure 6E). Amiloride inhibits exosome secretion [23] and is used to treat high blood pressure patients. To understand whether exosomes were involved in the regulation of MMP3 protein levels and MMP9 activities in tumor patients, we analyzed MMP3 protein levels and MMP9 activities in lung tumor tissues from obese tumor patients with or without amiloride treatment. Interestingly, we observed that MMP3 protein levels and MMP9 activity in patients with amiloride treatment were significantly reduced compared with patients without amiloride treatment (Figure 6F, 6G). Together, these data demonstrate that obesity is responsible for MMP3-induced MMP9 activation in tumor patients and this process may be mediated by exosomes.

## DISCUSSION

Although a positive correlation between obesity and tumor metastasis has been revealed, previous publications mainly focused on the role of obesity-related inflammation in the tumor metastasis [24–26]. In this study, we find that adipocytes are rich in MMP3 protein and can pass these proteins to their exosomes. After fusion with tumor cells, adipocyte-derived exosomes transfer MMP3 into tumor cells and enhance the invasive ability of tumor cells by activating MMP9. Therefore, our results indicate the important role of adipocyte-derived exosomes in obesity and tumor metastasis.

In addition, 3T3-A-EXO increased MMP3 protein level in 3LL tumor cells. However, treatment with the MMP3 inhibitor did not completely abrogate the increased invasive ability of 3T3-A-EXO-treated 3LL cells. This effect may be mediated by previously activated MMP9 by MMP3. In addition, MMP9 inhibitor treatment or MMP9 knockdown in 3LL tumor cells completely abolished 3T3-A-EXO-induced 3LL tumor cell invasion *in vitro* or *in vivo*, respectively. These results support the notion that MMP3 transferred by 3T3-A-EXO does not directly promote 3LL tumor cell invasion but requires the activation of MMP9. Although MMP3 mediates tumor metastasis [27–30], few publications suggest that MMP3 promotes tumor invasion by directly degrading the extracellular matrix. Given its specific role in activating pro-MMP-1, -3, -7, -8, -9 and -13 [31–33], the possibility that these effects are mediated by other MMPs activated by MMP3 cannot be excluded. Therefore, MMP3 likely weakly promotes tumor invasion by itself but mainly acts through activation of other MMPs, such as MMP9, in 3LL cells.

After inhibition of 3T3-A-EXO uptake by cytochalasin D, increased MMP9 activity in 3LL tumor cells could not be detected, suggesting that MMP3 in 3T3-A-EXO cannot directly induce MMP9 activation and fusion of 3T3-A-EXO and that 3LL tumor cells are required for this process. MMPs are subdivided into subgroups based on their substrate specificity and structural properties, including gelatinases (MMP-2 and 9); stromelysins (MMP-3, 10 and 11); collagenases (MMP-1, 8 and 13); matrilysins (MMP-7 and 26) and membrane-type MMPs (MMP-14, 15, 16, 17, 24 and 25) [34]. Because MMP3 is not membrane-type MMP, it must be packaged into exosomes. It is reasonable that 3T3-A-EXO must fuse with 3LL tumor cells. Then, MMP3 in 3T3-A-EXO is released to exert its functions.

Increased MMP3 protein levels were noted in tumor tissues from obese patients, and MMP3 protein levels were positively correlated with MMP9 activities. These results suggest that MMP3 is implicated in MMP9 activation in tumor tissues of obese patients. As an inhibitor of H^+^/Na^+^ and Na^+^/Ca2^+^ channels [35], amiloride inhibits exosome production [23]. In obese tumor patients treated with amiloride, increased MMP3 protein levels and MMP9 activities were detected in tumor tissues, suggesting that exosome-mediated MMP3 protein transfer and subsequent MMP9 activation may also occur in the human body. However, as a pleiotropic agent [36, 37], amiloride likely induces MMP3 protein levels and MMP9 activities via other mechanisms. Therefore, whether exosomes are involved in the upregulation of MMP3 protein levels and MMP9 activities requires further study.

In summary, MMP3 protein is enriched in 3T3-A-EXO and can be transferred into 3LL tumor cells by exosome and cell fusion. After transfer, MMP3 activates MMP9 in 3LL tumor cells, which improves the invasive ability of 3LL tumor cells. In addition, increased MMP3 protein levels and MMP9 activities are noted in tumor tissues from obese lung cancer patients, which may also result from exosome-mediating protein transfer. Thus, our findings decipher a novel mechanism for obesity and lung cancer metastasis, in which adipocyte-derived exosomes participate.

## MATERIALS AND METHODS

### Antibodies and reagents

Anti-CD63 (MX-49.129.5), anti-TSG101 (EPR7130(B)), anti-Alix (EPR15314), anti-GRP94 (9G10) and anti-Calnexin (AF18) antibodies were purchased from Abcam (Cambridge, MA, USA). Anti-MMP2 (10373-2-AP), anti-MMP3 (17873-1-AP), anti-MMP9 (10375-2-AP), anti-urokinase-type plasminogen activator (uPA) (17968-1-AP) and anti-Cathepsin B (12216-1-AP) antibodies were purchased from Proteintech (Rosemont, IL, USA). Cell Counting Kit-8 (CCK-8) solution was purchased from Dojindo (Tokyo, Japan). Dulbecco’s modified Eagle’s medium (DMEM), fetal bovine serum (FBS), dexamethasone, insulin, BCA protein assay, Matrigel matrix basement membrane and carboxyfluorescein diacetate succinimidyl-ester (CFSE) were obtained from Thermo Fisher Scientific (Waltham, MA, USA). Gelatin and 1-methyl-3-isobutylxanthine were purchased from Sigma-Aldrich (St. Louis, MO, USA). The MMP3 specific inhibitor UK 356618, MMP9 inhibitor I, cytochalasin D, cycloheximide and MMP-9 shRNA (m) lentiviral particles were purchased from Santa Cruz Biotechnology (Santa Cruz, CA, USA). Polyvinylidene fluoride membrane, enhanced chemiluminescence reagents and Amicon ultra centrifugal filters (50 KD) were purchased from Millipore (Billerica, MA, USA).

### Mice and cell lines

Female C57BL/6J and athymic nude mice (6-8 wk old) mice were purchased from Joint Ventures Sipper BK Experimental Animal Co. (Shanghai, China). Mice were housed in a specific pathogen-free facility, and the experimental protocols were approved by the Animal Care and Use Committee of School of Medicine, Zhejiang University (Hangzhou, China).

The mouse embryo 3T3-L1 cell line, mouse 3LL Lewis lung cancer cell line that originated from C57BL/6 mice and human A549 lung cancer cell line were purchased from the American Type Culture Collection (ATCC, Manassas, VA, USA). The 3T3-L1 cells were cultured in low-glucose DMEM media containing 10% FBS for 3 days for cell proliferation. To induce adipocyte differentiation, mouse 3T3-L1 cells were cultured in high-glucose DMEM containing 10% FBS, 0.5 mM 1-methyl-3-isobutylxanthine and 0.25 μM dexamethasone for 2 days. Then, 3T3-L1 adipocytes were sustained in high-glucose DMEM containing 10% FBS and 5 μg/ml insulin, and the medium was changed every other day [38].

### Human samples

Human tumor tissues from obese and non-obese patients were obtained from the Zhejiang Cancer Hospital (body mass index greater than 27 was considered obese). The collection of human samples was approved by the local Ethical Committee and the Review Board of the Zhejiang Cancer Hospital. All the participants were informed of the usage of the samples, and consent forms were obtained.

### Exosome isolation

The supernatant of 3T3-L1 or 3T3-L1 adipocytes was sequentially centrifuged at 300 × *g* for 10 min, 1,200 × *g* for 20 min and 10,000 × *g* for 20 min at 4°C. The supernatant from the final centrifugation was ultracentrifuged at 100,000 × *g* for 1 h at 4°C. By removing the supernatant, the exosome pellets were washed in a large volume of ice-cold PBS and centrifuged at 100,000× g for an additional 1 h at 4°C. Exosomes in the pellet were resuspended in PBS. Exosomes from 3T3-L1 cells or 3T3-L1 adipocytes were referred to as 3T3-EXO or 3T3-A-EXO, respectively.

### Electron microscopy

For electron microscopy observation, exosome pellets were fixed in 4% paraformaldehyde at 4°C for 1 h. Then, the pellets were loaded onto electron microscopy grids coated with formvar carbon, contrasted and embedded in a mixture of uranyl acetate and methylcellulose. Sections were observed with a Philips Tecnai-10 transmission electron microscope operating at 80 kV (Phillips Electronic Instruments, Mahway, NJ, USA).

### Western blot

Briefly, 3LL cells (1 × 10^6^/ml) were co-incubated with 30 μg/ml 3T3-EXO or 3T3-A-EXO with or without 10 μg/ml cycloheximide at 37°C for 4 h. Occasionally, 3LL cells were pre-treated with 10 μg/ml cytochalasin D for 30 min. Then, the cells were lysed by cell lysis buffer.

Protein concentrations of exosomes or cell lysates were determined using the BCA assay, and equal amounts of proteins (20 μg) were separated on a 10% SDS-PAGE and electrotransferred onto a PVDF membrane. The membranes were blocked in Tris-buffered saline Tween containing 5% fat-free dry milk and then incubated with the corresponding primary antibodies overnight at 4°C followed by incubation with a horseradish peroxidase-conjugated secondary antibody for 1 h. Proteins were detected using enhanced chemiluminescence reagents.

### Detection of cell growth *in vitro*

Five thousand 3LL cells were pretreated with 30 μg/ml 3T3-EXO or 3T3-A-EXO for 4 h, and then the cells were collected and re-cultured in fresh RPMI 1640 medium with 10% FBS for 24, 48 and 72 h. Twenty μl of CCK-8 were added per well 4 h before the end of culture, and the absorbance was detected at 450 nm.

### *In vitro* 3LL cell migration and invasion assay

For the migration assay, 3LL cells (1 × 10^6^/ml) were treated with 30 μg/ml 3T3-EXO or 3T3-A-EXO at 37°C for 4 h, and then 1 × 10^4^ 3LL cells were transferred into 100 μl of serum-free media and seeded onto the top of the Transwell chambers (8-μm pore size). For the invasion assay, Matrigel was rehydrated using a 6-fold volume serum-free media, and 50 μl rehydrated Matrigel at concentration of 200 μg/ml were added onto an 8-μm polycarbonate membrane in 24-well Transwell plates. Matrigel was solidified at 37°C. 3LL cells (1 × 10^6^/ml) were treated with 30 μg/ml 3T3-EXO or 3T3-A-EXO with or without 10 nM MMP9 inhibitor at 37°C for 4 h. In addition, 3LL cells were pre-treated with 10 nM UK 356618 for 2 h. Then, 2 × 10^4^ 3LL cells in 100 μl of serum-free media were seeded into the top chamber. The bottom chamber was filled with 800 μl of RPMI 1640 culture medium containing 20% FBS. After a 48-h incubation at 37°C, the cells were fixed with methanol for 20 min and washed three times with PBS for 20 min each. The fixed cells were stained with 10 mg/ml of DAPI for 30 min and washed with PBS. The stained cells were examined under a florescence microscope.

### Gelatin zymography

Briefly, 3LL cells (1 × 10^6^/ml) were co-incubated with 30 μg/ml 3T3-EXO or 3T3-A-EXO with or without 10 nM MMP9 inhibitor at 37°C for 4 h. In addition, 3LL cells were pre-treated with 10 μg/ml cytochalasin D for 30 min. Then, the cells were collected and cultured in serum-free RPMI 1640 media with or without 10 nM UK 356618 for an additional 24 h. Then, the supernatants were collected and concentrated with an Amicon centrifugal filter (50 KD) at 6,000 × *g* for 15 min. The concentrate was subjected to gelatin zymography assay.

Lung tumor tissues from patients of the same quality were mechanically homogenized in 1 ml PBS followed by centrifugation at 12,000 × *g* for 15 min. The supernatants were collected for gelatin zymography assay.

Gelatin zymography was performed as previously described [39]. In general, supernatants were diluted 1:1 in non-reducing sample buffer and separated by sodium dodecyl sulfate polyacrylamide gel electrophoresis (SDS-PAGE) using an 8% polyacrylamide gel containing 1.5 mg/ml gelatin for 60 min at 85 V followed by an additional 60 min at 130 V. The gel was renatured by incubation with 2.5% Triton X-100 for 30 min at room temperature. The gels were washed for 30 min in developing buffer (50 mM Tris-HCl pH 8.0, 2.5 mM CaCl_2_) overnight at 37°C. Finally, gels were stained with 0.25% Coomassie brilliant blue R-250 for 45 min and then destained appropriately. The area degraded by MMP9 was quantified using ImageJ 1.8.0 software (NIH, Bethesda, MD, USA).

### Inhibition of exosome uptake by 3LL cells

Here, 3T3-A-EXO CFSE was used as previously described [40]. Briefly, 3T3-A-EXO (20 μg) collected after 100,000 × *g* ultracentrifugation was incubated with 7.5 μM CFSE for 30 min at 37°C in a final volume of 200 μl PBS containing 0.5% BSA. CFSE-labeled 3T3-A-EXO were washed in a large volume of ice-cold PBS and centrifuged at 100,000 × *g* for an additional 1 h at 4°C. CFSE-labeled 3T3-A-EXO was resuspended in PBS and incubated with 3LL cells (pretreatment with 10 μg/ml cytochalasin D for 30 min at 37°C) for 4 h at 37°C. The uptake of 3T3-A-EXO was observed by confocal microscopy.

### RNA interference assay

For transient silencing of MMP3, the following 21-nt sequences of small interfering RNA (siRNA) duplexes were synthesized (GenePharma, Shanghai, China) were used: 5’-CAAGAUGAUGUAGAUGGUATT-3’ (sense) and 5’-AAUACCAUCUACAUCAUCUUG-3’ (antisense). In addition, 5’-UUCUCCGAACGUGUCACGUTT-3’ (sense) and 5’-ACGUGACACGUUCGGAGAATT-3’ (antisense) were synthesized as the negative control (NC) siRNA. Forty nM of siRNA duplexes were transfected into cells (2×10^5^/well) using 3 μl of INTERFER in siRNA transfection reagent (Polyplus, NY, CA, USA) in 24-well plates. The efficiency of transient MMP3 silencing was confirmed by Western blot.

### Establishment of 3LL cells with stable MMP9 shRNA expression

Briefly, 3LL cells were transduced with MMP-9 shRNA (m) lentiviral particles to silence MMP9 or with negative control particles according to the manufacturer’s instructions. After infection by lentiviral particles, 3LL cells were cultured in the presence of 1 μg/ml puromycin. The media was changed every 3 to 4 day, and puromycin-resistant cell clones were selected after assessing MMP9 downregulation by Western blot.

### Lung metastasis assay *in vivo*

Briefly, 3LL cells or 3LL cells with MMP9 knockdown (1 × 10^6^ per mouse) pre-treated with 30 μg/ml 3T3-EXO or 3T3-A-EXO for 4 h were injected into 6-wk-old C57BL/6J mice via the tail vein. Fifteen days after cell injection, the mice were euthanized. A549 cells (2 × 10^6^ per mouse) pre-treated with 30 μg/ml 3T3-EXO or 3T3-A-EXO for 4 h were injected into 6-wk-old nude mice via the tail vein. Twenty-four days after cell injection, the mice were euthanized. The lungs were removed, and the numbers of lung tumor foci were counted under a dissecting microscope. The weights of lungs were also measured.

### Immunohistochemistry

The lung tumor tissues from patients were fixed in 10% formalin, dehydrated in ethanol, and embedded in paraffin. Tissue sections were cut at 4 μm, mounted on slides and dried at 60°C for 4 h. Following short proteolytic digestion and a peroxidase block of the tissue slides using 2.5% hydrogen peroxide in methanol for 30 min at room temperature, the slides were incubated with the anti-MMP3 antibody overnight at 4°C. After washing, the slides were incubated with peroxidase-labeled polymer and substrate chromogen. Finally, the specimens were incubated in phosphate buffered saline containing diaminobenzidine for 5 min. An Olympus microscope was employed to visualize the staining of the tumor tissues. Images of the sections were captured, and positive areas were analyzed. The quantity of MMP3 was calculated as the mean density defined as the integrated optical density divided by the actual area.

### Statistical analysis

The data are presented as the mean ± SEM. Comparisons between two groups were performed using Student’s *t*-test. Comparisons between multiple groups were performed using one-way ANOVA and Newman-Keuls test, and the Spearman rank order correlation test was used to examine correlations between MMP3 protein levels and MMP9 activities in lung tumor tissues from patients using GraphPad Prism 5 (San Diego, CA, USA). Statistical significance was determined at *p* < 0.05.

## SUPPLEMENTARY MATERIALS FIGURES


