# Supplementary material for: Adipocyte-derived exosomes promote lung cancer metastasis by increasing MMP9 activity via transferring MMP3 to lung cancer cells
Source: Oncotarget. 2017 Jun 27;8(47):81880–91. doi: 10.18632/oncotarget.18737 (PMC5669856; doi:10.18632/oncotarget.18737)
Supplement: Supplementary file 1 [file oncotarget-08-81880-s001.pdf]

Adipocyte-derived exosomes promote lung cancer metastasis by increasing MMP9 activity via transferring MMP3 to lung cancer cells

SUPPLEMENTARY MATERIALS

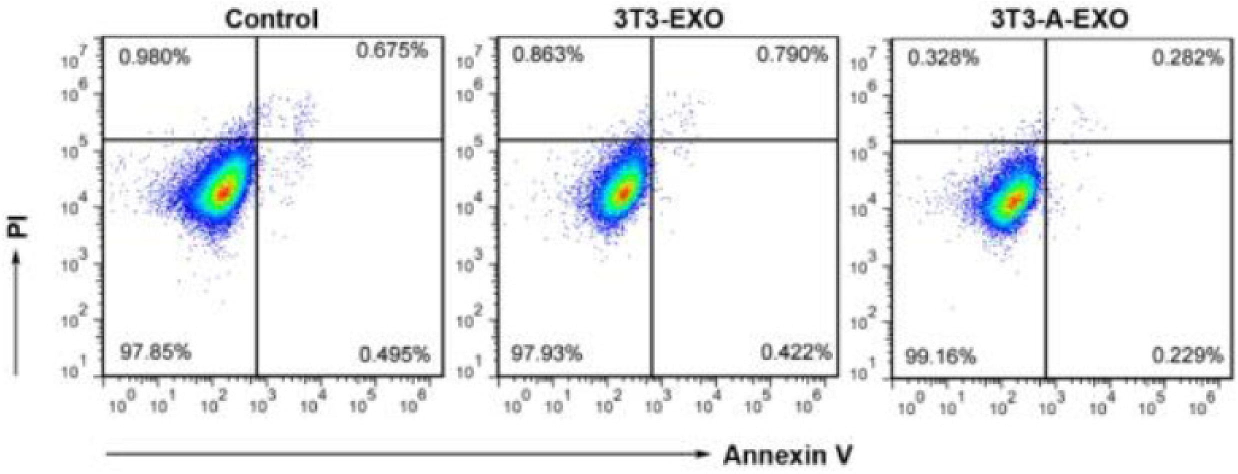

Supplementary Figure 1: Adipocyte-derived exosomes did not affect 3LL cell apoptosis. 3LL cells were treated with 30 µg/ml 3T3-EXO or 3T3-A-EXO for 24 h. Then the cells were collected and stained with Annexin V and PI. The apoptosis of cells was analyzed by flow cytometry. One representative data from three independent experiments was shown.

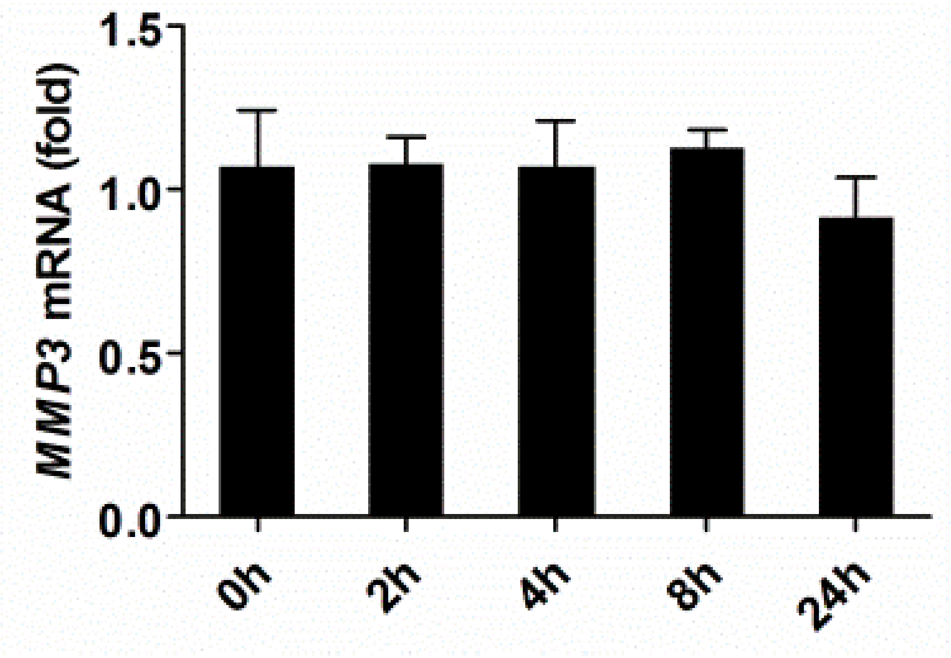

Supplementary Figure 2: Kinetics of *MMP3* mRNA in 3T3-A-EXO-treated 3LL cells. 3LL cells were treated with 30 μg/ml 3T3-A-EXO for 4 h, and then *MMP3* mRNA levels were detected by real-time PCR at different time points. The data are representative of two independent experiments.

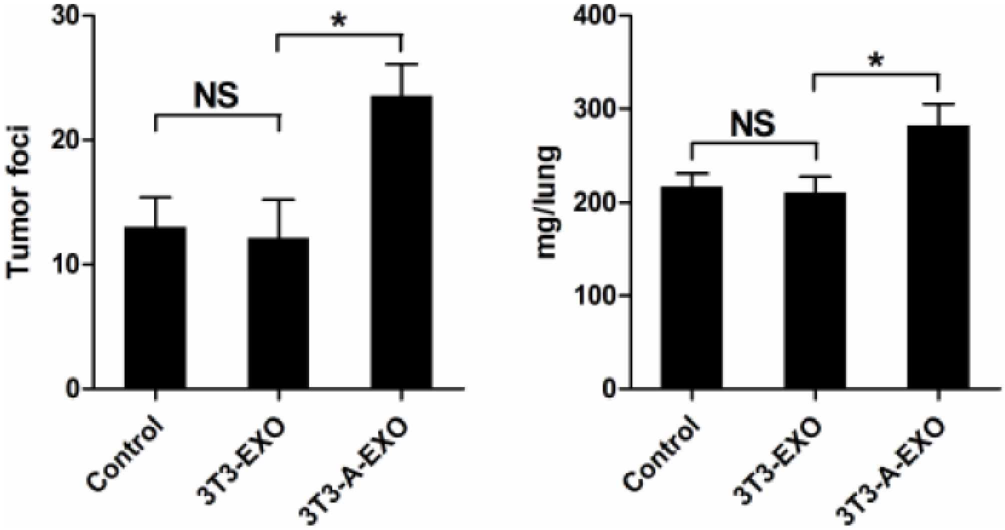

Supplementary Figure 3: 3T3-A-EXO promotes A549 tumor cell metastasis *in vivo*. (A, B) A549 cells were treated with 30 µg/ml 3T3-EXO or 3T3-A-EXO for 4 h. Then, 2×10^6 tumor cells were intravenously injected into nude mice, and these mice were euthanized 24 days later. Lung tumor foci were statistically analyzed (n = 5) **(A)**. The weights of lungs were statistically analyzed (n = 5) **(B)**. **(A, B)** the results are shown as the mean ± SEM of three independent experiments. *P*-values were generated by one-way ANOVA, followed by Tukey-Kramer multiple comparisons test; $*p< 0.05$; NS, not significant. Control indicates 3LL cells treated with PBS.
